# Nutrient Digestibility and Retention of Potential Feed Ingredients for Rainbow Trout (*Oncorhynchus mykiss* W.) Aquaculture in Iran

**DOI:** 10.1155/2023/8910005

**Published:** 2023-12-20

**Authors:** Hamed Salehi, Stefan Reiser, Ulfert Focken

**Affiliations:** Thünen Institute of Fisheries Ecology, Herwigstr. 31, 27572 Bremerhaven, Germany

## Abstract

To overcome the aquafeed challenges for aquaculture in Iran, the world's largest rainbow trout producer, the nutrient digestibility and retention of eight rendered poultry by-products, and two plant protein sources were investigated. The test ingredients were added to a casein-based semipurified reference diet as control diet and fed to juvenile rainbow trout in three runs. During the experimental runs, test ingredients were added to the control diet in a ratio of 30 : 70. The diets were then fed to the animals in four replicates. TiO_2_ was used as marker and feces were collected by settling method. The apparent protein digestibility coefficient for poultry by-product meals (PBM), feather meals (FeM), poultry protein concentrate (PPCon), blood meal (BM), canola meal (CM), and Iranian soybean meal (IRSBM) were 73%–93%, 73%–96%, 100%, 87%, 94%, and 97%, respectively. The nitrogen productive value for PBM ranged 27%–46%, both PPCon and IRSBM 37%, CM 24%, BM 23%, and FeM from −6% to 18%. Citric acid supplementation of the alkaline thermochemically hydrolyzed FeM improved the digestibility of crude lipid and organic matter from 6% and 88% to 55% and 92%, respectively. The assessment of digestibility as well as nutrient retention analysis for tested feed components indicated that refined PBM and PPCon, both CM and IRSBM could be used in rainbow trout diets as value-added and eco-friendly protein sources. The findings of this study can assist fish nutritionists and key players in the aquafeed industry in adopting a sustainable approach to aquaculture by selecting locally available raw materials that demonstrate high nutrient retention in rainbow trout.

## 1. Introduction

Aquaculture is currently recognized as the fastest-growing food provider globally. Between 2018 and 2020, the cultured aquatic animals experienced a consistent annual average growth rate of 3.4%. The production volume for that sector has increased from 82.5 million tons in 2018 to 90.9 million tons in 2021, reflecting a significant double-digit growth rate of 10.2% during this period [1]. In 2020, aquaculture accounted for a significant share of the world's aquatic food supply, contributing to approximately 56% of the total production which was greater than 52% in 2018 [2, 3]. This highlights the increasing importance and contribution of aquaculture in meeting global food demands. It is anticipated that the aquaculture production of high-value species like shrimps, salmon, and trout will continue to increase by 2030. However, an important consideration is the availability of fish meal, which serves as a vital feed ingredient for these species. Projections suggest that fish meal production will only experience a slight increase by 2030, falling significantly short of the expected rise in demand for fish and seafood [4]. This situation poses a potential challenge in meeting the growing feed requirements of valuable aquaculture species. As of 2021, Iran held the distinction of being the prominent global producer of rainbow trout with a production volume of approximately 194,000 tons. The country accounted for approximately 20.3% of the total global production of finfish [1]. In view of the status of wild fish stocks and the protein requirements of the growing aquaculture industry in Iran and the world, there is a pressing need for additional sustainable and readily available protein sources for the aquafeed industry. These sources should not compete with human nutrition but represent an actual net gain. Meanwhile, aquaculture utilizes water resources in a nonconsumptive manner but adds nutrients originating from the feed to the aquatic environment which can contribute to eutrophication. One of the crucial approaches in order to minimize eutrophication risk and water pollution is producing aquafeeds with highly digestible feed ingredients [5]. Thus, digestible feed components with high nutrient retention rates are required to safeguard sustainable growth.

To identify promising feed components for a sustainable growth of rainbow trout production, exploring renewable and abundant local resources is crucial. Despite possessing a favorable proximate composition for fish nutrition, including a protein content of 41%, lipid content of 4%, fiber content of 12%, and ash content of 7.3% on a dry matter (DM) basis [6], canola meal (CM) is still not widely recognized as a popular commodity for feeding fish in Iran due to presence of antinutrients in rapeseed cultivars [7, 8]. In 2021, the production of canola seed reached approximately 215,000 tons, exceeding the production of soybean (200,000 tons) and other primary oilseeds [9]. Through the process of oil extraction, around 118,000 tons of CM or cake can be obtained from that harvested canola seed, which accounts for around 55% of the total yield. Hence, considering its substantial production and suitability, CM could serve as a dependable plant-based protein source for salmonid species in Iran's future aquaculture. Moreover, the poultry industry holds significant importance in supplying protein to the population of Iran. According to FAO, Iran produced 1,983,328 tons of chicken meat in 2021 [9]. Assuming an average dressing percentage of 70% for chicken, this suggests that approximately 2,833,300 tons of live chicken were produced in that year [10]. This production yielded around 198,300 tons raw feather (7%), 99,165 tons fresh blood (3.5%), and 495,827 tons of other remains (17.5%). The feed compositions exhibit significant variations across different sources and batches, especially concerning rendered animal protein sources derived from various processing techniques [6, 11]. These variations result in nutritional disparities among rendered poultry protein ingredients. Moreover, due to the limited availability of processing and refining technology, certain components of these by-products are currently not regarded as high-quality aquafeed sources in Iran [12]. Soya and soybean meal were the main imported plant protein sources in recent years [13]. Although locally produced soybean cannot fulfill the requirements of aquafeed market, the digestibility and nutritional value for this strategic commodity need to be tested in rainbow trout.

In the current study, not only the digestibility of some potential plant and animal protein feed components for the aquafeed industry in Iran was taken into consideration but also studied for the improvement of the digestibility of a locally produced feather meal by means of dietary acidification for rainbow trout nutrition. In addition to the digestibility analysis, the share of nutrient retention from the total diet that can be attributed to the tested ingredients has been calculated by comparative slaughter method and digestibility formula which is a novel approach. In the first run, the digestibility of three different feather meals (FeM), including regular feather meal (RegFeM), GOLDMEHL® feather meal (GoldFeM), Iranian feather meal (IRFeM), a CM, and a poultry by-product meal (PBM), containing minimum 64% crude protein (PBM_64) were examined. The test diet containing IRFeM was treated with citric acid and tested in the second run for a comparison to the attributed observations in the first run. In the third run, a local Iranian soybean meal (IRSBM), a poultry protein concentrate (PPCon), an ultra-flash dried poultry blood meal (BM), and two different poultry by-products meals containing maximun 11% ash content as low-ash (PBM_LA) and with minimum 50% crude protein (PBM_50) were assessed. This study represents the first attempt to evaluate the apparent nutrient digestibility coefficients for thermochemically processed FeM, PPCon, and ultra-flash-dried BM in rainbow trout using the settling feces collection method. The findings of this study provide valuable datasets that can be used to produce high-quality commercial rainbow trout feed in Iran, comparable to what is in Germany. Access to locally sourced feed components that have high digestibility is crucial for the development of sustainable and environmentally friendly feed options in salmonid production worldwide.

## 2. Materials and Methods

### 2.1. Diet Preparation

All of the animal test ingredients excluding the IRFeM were provided by GePro (Geflügel-Protein Vertriebsgesellschaft mbH & Co. KG) and its poultry processing unit (A&L Tierfrischmehl Produktions GmbH) in Diepholz, Germany. The CM was purchased from an oil mill in Germany (Teutoburger Ölmühle GmbH, Ibbenbüren, Germany) but the IRSBM was produced in Iran. The IRFeM tested in runs 1 and 2 was processed chemically (NaOH [0.5 M], soaked 2 hr at room temperature) and autoclaved (127°C, 90 min) at the Poultry Science Department Faculty of Agriculture, Tarbiat Modares University, Tehran, Iran. A casein-based semisynthetic laboratory standard diet (modified Guelph Test Diet) for the digestibility assessments was produced as the reference diet [6, 14, 15]. This diet was formulated to meet the recommendations of NRC [6] for the nutrition of juvenile rainbow trout. To neutralize its low pH, some sodium carbonate (Na_2_Co_3_) was added to its formulation in run 3.

All solid feed components with coarse particles, except for the BM and PPCon (AquaTrac® sol SD), were ground and sieved through a 2 mm screen. The test diets contained 700 g kg^−1^ of the reference diet and 300 g kg^−1^ of the test ingredients on the basis of DM (Table 1). Titanium (IV) oxide (Sigma–Aldrich Chemie GmbH, Steinheim, Germany) was added as an indigestible marker to all diets. All of the dry components were added to a kitchen blender in an ascending order of volume and mixed thoroughly. Oils were added toward the end of the mixing process. An amount of 15% distilled water was added to the experimental mixtures and gradually increased to a maximum of 35% until reaching the right consistency of the resultant paste. The prepared mixtures were pelleted with a meat mincer passed through a die with holes of 4 mm diameter. Produced strands were chopped into pieces that could easily be ingested by the respective fish size and dried in an electronic oven (T 12, Thermo Electron LED GmbH, Langenselbold, Germany) at 30°C for 24 hr. The dried products were packed in airtight buckets and stored at 4°C until use.

### 2.2. Fish Rearing

The experiments were conducted in a windowless and temperature-controlled room at the Thünen Institute of Fisheries Ecology in Bremerhaven, Germany. Juvenile rainbow trout (*Oncorhynchus mykiss* W.) used for the experiments originated from a broodstock kept at the institute. Before conducting every run of the experiment and upon stocking in the experimental units for acclimation, the fish were fed with a commercial feed (ALLER FUTURA 2 mm, Emsland-AllerAqua GmbH, Golßen, Germany). The fish for every diet were randomly allocated to four 57-l aquaria. The aquaria had inclined base plates and were connected to a semirecirculating aquaculture system. The system was oxygenated with an air compressor and equipped with pad filter, ultraviolet (UV) light, biofilter, and a water temperature control device. Water inflow to every aquarium was adjusted to keep dissolved oxygen and water temperature within the optimal range and in order to allow sedimentation of feces. Oxygen concentration (mg L^−1^) was measured with probe daily from inflow and outflow water basins 2 hr after feeding and weekly within all aquaria. Water pH was determined by probe but NH_4_^+^, NO_2_^−^, and NO_3_^−^ were measured photometrically (Spectroquant® test kit, Merck KGaA, Darmstadt, Germany) every 2 days from the inflow water basin. An overview of the experimental conditions is given in Table 2. All experimental procedures were conducted in accordance with European Directive 2010/63/EU on the protection of animals used for scientific purposes.

### 2.3. Feeding Procedure

Fish were weighed individually at the beginning and at the end of the trails as well as every second week. Fish were starved 24 hr before weight determination. Right after stocking and at the beginning of the trials, fish were fed at 0.5% biomass weight during the short acclimation. Feeding was increased gradually by offering the experimental diets to the level of maximum 2% biomass weight per day as soon as the feed was fully accepted by the fish. The fish received diets in four replicates (*n* = 4) in a randomized block design. The daily feed ration was divided into two equal installments and were hand-fed at 9:00 and 14:00 to prevent any feed losses. Feed ingestion by the fish was observed and stopped in case the fish refused intake and the rest of the feed was recorded. Rare cases of mortality were also recorded and frozen immediately.

### 2.4. Sample Collection, Sample Preparation, and Chemical Analyses

For the comparative slaughter method analyses and at the beginning of each run, some fish were randomly selected as reference from the batch of fish used for the experiments. Fish were anesthetized with 2-phenoxyethanol (0.5 mL L^−1^, Merck KGaA, Darmstadt, Germany), weighed and sacrificed by cutting the gill artery and stored at −21°C until further preparation. At the end of each run, the identical procedures were followed. In order to prepare the fish samples for analysis, fish were defrosted overnight at 4°C and subsequently chopped and autoclaved at 121°C and 210.2 kPa for 5 min to have a homogenized mix after grinding. The homogenized samples were frozen at −21°C for minimum 48 hr prior to freeze drying (Lyo GT, SRK Systemtechnik GmbH, Riedstadt, Germany). After that, the fish samples were ground again.

Feces were collected after the fish were fed the experimental diets for at least 7 days. Excreta were gently siphoned from the bottom of each aquarium three times daily. Excreta were sampled before offering the feed in the morning and 2 hr after feeding to prevent any nutrients from leaching. Feces collected from each aquarium were pooled and frozen at −21°C and later dried as described for the fish samples. The dried excreta were pulverized and sieved with a 0.5 mm screen. The experimental diets were also ground and passed through a 1.0 mm sieve. The prepared feed, feces, and fish samples were stored in sealed plastic containers at −21°C until further lab analyses.

The proximate composition analyses were conducted based on the official methods in Germany [16]. To measure DM content, the samples were dried in an electric oven at 103°C for 4 hr. The samples were burned in a muffle furnace at 550°C for 3 hr in order to conduct ash content analysis. These parameters were measured in triplicate. To measure gross energy, a bomb calorimeter was used and samples were tested in duplicate (C2000, IKA-Werke, Staufen, Germany). Crude fiber (CF) contents in experimental diets as well as trypsin inhibitor activity in IRSBM were analyzed by AGROLAB LUFA GmbH (Kiel, Germany) according to the European Union Regulations (EC/No 152/2009). Crude protein (CP) and crude lipid (CL) in the fish samples were determined by LABOR IBEN GmbH (Bremerhaven, Germany) based on ASU L 06.00-7 2014-08. By subtracting determined CP, CL, CF, and ash from 1,000, the nitrogen-free extract (NFE) for the diets was obtained. The organic matter (OM) content was also calculated as the difference between the ash content and 1,000. The minerals as well as TiO_2_ were determined as described in detail by Zeller et al. [17] in the Institute of Animal Nutrition at the University of Hohenheim, Stuttgart, Germany. The pH in experimental diets was measured with a probe (Type 7110, Xylem Analytics Germany GmbH, Weilheim, Germany). The analytical data for ingredients and test diets are presented in Tables 3 and 4, respectively.

### 2.5. Calculations

To calculate the apparent digestibility coefficients (ADCs) of the nutrients like CP, CL, and OM in diets and test ingredients, the following recommended formulae by NRC [6] were used. The formula for measuring nutrient digestibility was also used to calculate the nutrient productive values for each ingredient:

(1) $$\begin{array}{c} {\text{ADC}}_{\text{test diets}}=\left(1-\left({\text{TiO}}_{2}\text{ concentration in feed}\right)/\left({\text{TiO}}_{2}\text{ concentration in feces}\right)\times \text{(Nutrient concentration in feces)}/\text{(Nutrient concentration in feed)}\right)\times 100, \end{array}$$

(2) $$\begin{array}{c} {\text{ADC}}_{\text{test ingredients}}={\text{ADC}}_{\text{test diet}}+\left[\left({\text{ADC}}_{\text{test diet}}-{\text{ADC}}_{\text{ref diet}}\right)\times \left(0.7\times D_{\text{ref}}/0.3\times D_{\text{ingredient}}\right)\right]. \end{array}$$

where *D*_ref_ is the percentage of nutrient in the reference diet and *D*_ingredient_ is the percentage of nutrient in the ingredient.

To assess the retention of the nitrogen and lipid from the experimental diets in the fish, the nitrogen productive value (NPV) and lipid productive value (LPV) were calculated with the nutrient productive value (NutrPV) formula:

(3) $$\begin{array}{c} \text{NutrPV}\left(\%\right)=\left(\left(\text{Final fish body nutrient in g - Initial fish body nutrient in g}\right)/\text{Total consumed nutrient in g}\right)\times 100. \end{array}$$

The NutrPV_test ingredient_ was calculated analogous to ADC_test ingredient_, as follows:

(4) $$\begin{array}{c} {\text{NutrPV}}_{\text{test ingredients}}={\text{NutrPV}}_{\text{test diet}}+\left[\left({\text{NutrPV}}_{\text{test diet}}-{\text{NutrPV}}_{\text{ref diet}}\right)\times \left(0.7\times D_{\text{ref}}/0.3\times D_{\text{ingredient}}\right)\right], \end{array}$$

where nutrient is either nitrogen (N) or CL and the initial fish nutrients refer to those nutrients in the reference fish which were slaughtered at the beginning of each run.

In order to assess the influence of citric acid on the high pH value of IRFeM, the measured factors regarding that ingredient in both the first and second runs were divided into the average value of that parameter for the reference diet in each run and then multiplied by 100 to get the relative values.

### 2.6. Statistical Analysis

Statistical analysis was conducted by using the arithmetic means of traits analyzed. To assess the effect of citric acid treatment on the IRFeM diet, the relative values of that diet to the reference were compared between runs 1 and 2. Before analysis, Shapiro–Wilk test was used to evaluate the normality for all parameters separately in all runs. The Levene test was used to assess for homogeneity of variance for all parameters in experimental runs 1 and 3, but the *F*-test for run 2. Student's *t*-test was used when normality and homogeneity of variance were met otherwise Welche's *t*-test was applied to compare the mean values of two data sets for run 2. It was also analyzed whether the individual aquarium should be added as a random effect. Since the aquarium did not influence significantly, a linear model without random component was run, and one-way ANOVA was conducted for comparing the mean values in runs 2 and 3. Kruskal–Wallis test was conducted when normality assumption was violated. To identify the pairwise differences between means in runs 1 and 3, Tukey's HSD and Dunn's tests were carried out for parametric and nonparametric variables, respectively. A significant level at *p* < 0.05 was assumed for the results. All data were analyzed statistically using R software version 3.5.1 [18].

## 3. Results

**Figure 1:**
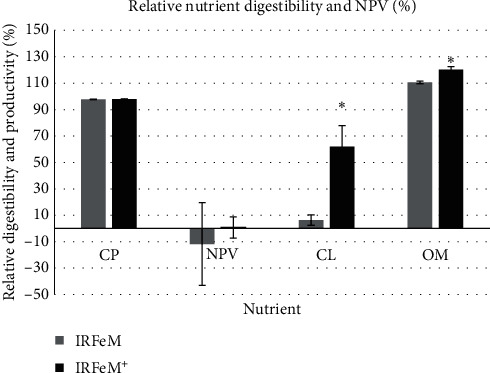
Relative nutrient digestibility and productivity (%) for Iranian feather meal. CP, crude protein; NPV, nitrogen productive value; CL, crude lipid; OM, organic matter; IRFeM, Iranian feather meal; IRFeM^+^, Iranian feather meal diet treated with citric acid (dissolved 0.02 g citric acid, C_6_H_12_O_6_, per 1 g Iranian feather meal and sprayed on IRFeM diet or 0.6% diet). The values are the mean of four replicates (*n* = 4). Error bars represent standard deviations from four replicate aquaria. Data with asterisks are considerably different (*p* < 0.05).

The retention and ADCs values for the nutrients measured in test diets and test ingredients are given in Table 5. The nutrient digestibilities and nitrogen productivity values in unsupplemented and supplemented IRFeM diet with citric acid relative to those parameters in the reference diets in runs 1 and 2 are presented in Figure 1.

Regarding the ADCs of CP, above 70% digestibility was observed for all the tested ingredients in all runs. In the first run, the IRFeM demonstrated a similar CP digestibility to other tested ingredients but higher value than the RegFeM. CP digestibility for CM (94%) and IRFeM (96%) was significantly higher than for RegFeM (73%), the other ingredients had intermediate values. The relative protein digestibility value for IRFeM remained unchanged in both runs 1 and 2. In run 3, PPCon and IRSBM had significantly higher digestibilities than the PBM_50; the CP ADC for BM differed considerably from PPCon in that run.

In terms of NPV, most ingredients did not differ significantly, except the IRFeM and PBM_64 having lowest and highest values, respectively. Comparing the relative NPV showed a nonsignificant improvement for the citric acid supplemented diet with IRFeM in run 2. The NPV for the tested ingredients in run 3 ranged from 23% for BM to 40% for PBM_LA.

Considering CL ADCs for the tested ingredients, the minimum value (6%) was noted for IRFeM in contrast to CM with the complete digestibility in run 1. By comparing the relative CL ADCs for IRFeM in runs 1 and 2, that nutrient was digested more effectively via supplementing citric acid (Figure 1). In run 3, a very high CL ADC (99%) was observed for PBM_LA, which was considerably higher (*p* < 0.05) than that nutrient digestibility for BM with a negative value. Regarding LPV for the ingredients, only the tested PBMs and PPCon showed the values either equal or higher than 50%. This parameter was also positive but lower than 25% for the tested CM and IRSBM. The negative LPV, furthermore, for the examined BM and FeMs was observed.

The OM ADC in tested ingredients ranged between 58% for RegFeM in run 1 to 100% for PPCon in run 3. Supplementing the diet containing IRFeM with citric acid in run 2 improved the OM ADC (Figure 1). In run 3, the maximim (100% ± 0.72%) and least (73% ± 1.60%) OM ADCs were observed for PPCon and PBM_50, respectively, and with intermediate values for the other tested ingredients.

## 4. Discussion

Due to systematic differences between digestibility values obtained from different methods of feces collection (stripping, settling), data from this study were compared only to other studies using settlement method for collecting feces as well. Exceptional cases are mentioned in the text.

### 4.1. Digestibility of Crude Protein

CP was digested between 73% and 96% in the three tested FeMs including RegFeM, GoldFeM, and IRFeM. RegFeM and IRFeM had the lowest and highest digestibility coefficients for protein, respectively. Both RegFeM and GoldFeM were steam hydrolyzed, but the former was dried with a disc dryer and the GoldFeM with a specific air drying system. Even though the protein content of GoldFeM was digested better than RegFeM, this difference was not statistically (*p* > 0.05) significant. Chemically processed IRFeM with sodium hydroxide had a CP ADC of 96% which was higher than the RegFeM but statistically not different from the GoldFeM. Protein quality in FeMs can be influenced greatly by processing. Both overcooked and undercooked feather meals have low protein digestibility [19]. The observed ADC of protein (73%) for the RegFeM was in line with Cheng et al. [20] who measured 77% CP digestibility for FeM in rainbow trout. Hajen et al. [21] also reported a CP ADC of 71% for a hydrolyzed FeM fed to postjuvenile chinook salmon (*Oncorhynchus tshawytscha*) in sea water. The values for the GoldFeM (88%) and IRFeM (96%) were in agreement or even higher than the previous studies. Sugiura et al. [15] reported 86%, Bureau et al. [11] observed values of 81% and 87%, Sugiura and Hardy [5] reported 83% and 86% ADC for CP. Although not statistically significant, a higher CP ADC for GoldFeM, indicating a better thermal hydrolyzation through two stages of drying processes (80 s at 270°C, 120 s at 80°C) rather than RegFeM (60 min 160°C). The sodium hydroxide (NaOH) processing and autoclavation have already demonstrated an increased of *in vitro* pepsin digestibility [22]. Adler et al. [23] predicted an *in vitro* protein digestibility of 90% in FeM treated with NaOH and autoclavation; however, a loss of cystine was observed. IRFeM represented a very good protein digestibility, but as the content of serine, cysteine, threonine, and arginine is substantially lower in that FeM than in the other FeM (Table 3), the effect of processing on the amino acid profile should be considered in the future. Neither the addition of citric acid to the alkaline diet containing IRFeM in run 2 nor the addition of Na_2_Co_3_ to the acidic reference diet in run 3 had significant effects on the protein digestibility (Table 5, Figure 1). Protein digestibility in rainbow trout might thus not depend primarily on the pH of the feed.

The CP ADCs for the different types of PBM were 73%, 89%, and 93% for PBM_50, PBM_64, and PBM_LA, respectively. The observations of this study are in line with the previous work on rainbow trout [5]. The observed apparent CP digestibility for PBM_50 was near to the reported value of 74% by Hajen et al. [21] for a PBM in chinook salmon. The values associated with the PBM_64 and PBM_LA were comparable to the works of Sugiura et al. [15] with 96% and Bureau et al. [11] with 87% and 91% for two PBM and Sevgili et al. [24] with 81% in rainbow trout. Furthermore, CP ADCs of 83%, 85%, and 87% were also reported for PBMs in that fish species by using settling methods [25]. All of the tested PBMs in our study were processed identically in terms of cooking and drying. They were dried with a disc dryer under 105°C for 40–60 min. The variation in ash content depends on the segregation of the raw materials used for processing. PBM_50 contained mostly turkey bones, whereas PBM_64 and PBM_LA had higher content of soft tissues. Some researchers already mentioned that protein digestibility can be negatively influenced by high ash content [21, 26]. However, the current study did not fully confirm this. According to Sugiura and Hardy [5], the main quota of ash content in animal-rendered products is calcium and phosphorous. By comparing mineral composition of tested PBMs (Table 3), it seems that higher calcium and phosphorous content may negatively affect the protein digestibility in rainbow trout. The tested IRFeM supports this hypothesis. IRFeM had the highest ash content compared to other feather meals but resulted in a better digestibility since the dominant mineral in its ash content is sodium from NaOH during chemical processing as also mentioned by Adler et al. [23].

PPCon showed an apparent CP digestibility of 100%. According to the producer, this product is made of the remained stickwater from poultry meal production which is then spray dried. The DM-based protein content of the tested PPCon (74.5%) was in accordance with chicken concentrate (76%) and the freeze-dried stickwater samples from fish processing industries (70.5% and 86.2%) [27, 28]. Bechtel [28] reported an *in vitro* protein digestibility of more than 95% using the 0.2% pepsin digestibility protocol. According to the study, this high digestibility could be due to the high water solubility of its proteins. Within feed manufacturing, the PPCon showed stickness properties and it was very palatable to the fish during our feeding experiment. Hence, PPCon might not only be added to fish diets as a pellet binder, but as a feed stimulant, especially in plant-based diets. The observed CP ADC for ultra-flash-dried BM in our study was 87% which was almost similar to the tested ring-dried BM (88%). However, it was higher than a rotoplate-dried (82%) and lower than spray-dried meals (96% and 97%) in rainbow trout [11]. Another work with chinook salmon showed a CP ADC of 29% for dried BM with a continuous dryer [21]. According to these authors, processing blood with excessive thermal procedure caused a low protein digestibility.

In the present study, 94% CP ADC was obtained for a feed grade CM which was higher than those already reported. CP digestibility of 91% and 89% for solvent-extracted and heat-treated CMs, respectively, reported in rainbow trout [29]. Moreover, in chinook salmon, an apparent CP digestibility of 85% was reported [21]. In this study, the observed high CP ADC for the feed grade CM may have been the result of genetic improvement of the plant and/or processing that increases digestibility.

IRSBM represented the CP ADC of 97%. This value was in accordance with the previous works on rainbow trout. Kaushik et al. [30] measured the CP digestibility of 93%, Sugiura et al. [15] measured a value of 90%, and Glencross et al. [31] reported 99% nitrogen digestibility in that fish species. The CP digestibility of IRSBM was notably high and it confirms the inefficacy of trypsin inhibitor in properly thermal treated soybean meals for fish as previously reported [32]. Trypsin inhibitor activity for the tested IRSBM was 5 mg g^−1^ DM and this is supposed to be not problematic for the rainbow trout with the average weight of 12.0 ± 0.9 g. The inclusion of that component in the experimental diet was only 30% on DM basis and that amount of trypsin inhibitor was therefore further diluted. According to Francis et al. [33], most of the cultured fish could tolerate up to 5 mg g^−1^ trypsin inhibitor.

### 4.2. Digestibility of Crude Lipid

The CL ADC in FeM and BM may not have primary importance for fish nutritionists because of low lipid content. However, a range of 6%–39% CL ADC was determined for tested FeMs in this study. Except GoldFeM with 39% ADC which was close to the result for a disc-dried tested FeM (40%) by Bureau et al. [11], the other tested FeMs in the current study showed lower values than in previous studies [11, 20]. Type of lipids and fatty acids in FeM may influence apparent lipid digestibility coefficients. High-quality FeMs should have a lipid content of less than 5%. Higher values indicate that feathers were contaminated with skin residues [19]. Saturated and monounsaturated lipids from skin residues might not be as digestible as unsaturated fatty acids. Moreover, FeM contains waxes which are resistant to degradation and utilization in animals because of including a long-chain fatty acid associated with a high-molecular-weight-monohydric alcohol [34]. Although wax esters could be hydrolyzed by fish lipases, this digestion is not as efficient as that of triacylglycerols [35]. The hydrolyzation degree of lipids in Atlantic salmon (*Salmo salar*) has been ordered as: triacylglycerol > wax esters > sterol esters [36]. The IRFeM diet treatment with citric acid increased the CL digestibility of that ingredient from 6% to 55%. This considerably improved CL ADC may be concluded from 0.5 unit diet acidification by citric acid. Bile salt-dependent lipase is more important to digest lipids in fish as mentioned by Bogevik et al. [36]. Tocher and Sargent [37] assessed the pH dependency of different bile salt-dependent lipases in rainbow trout and observed that the optimum pH for wax ester hydrolase activity was less than that for triacylglycerol and sterol hydrolases. The study also stated that though the pH value for treated IRFeM was more acidic (pH = 6.5) than the optimum in the *in vitro* study of those researchers (pH = 7.4), it seems that more wax esters from FeM were digested when this pH manipulation was applied.

A CL digestibility from 87% to 99% was observed in the tested PBMs. The lipid in the PBM_64 and PBM_LA was digested most efficiently with the values 98% and 99%, respectively. Observations in this study were comparable to one of the tested poultry meals (92%) from Bureau et al. [11], higher than all the tested PBMs (80%–83%) by Cheng and Hardy [25] and in line (93%) with Sevgili et al. [24]. The CL ADC and LPV for PPCon were 95% and 72%, respectively, indicating the high lipid bioavailability of that ingredient for rainbow trout. This lipid deposition was comparable to that lipid productivity (85%) for PBM_LA. The PPCon is a relatively new feedstuff in the aquafeed industry and there is no comparative data for it in fish yet. In view of the very low lipid content of 19 g kg^−1^ in BM, its digestibility is of minor importance. The obtained negative CL ADCs might be attributed to the very low lipid content of the investigated BM rather than that individual nutrient in the reference diet and this affected negatively on the lipid digestibility of BM. Since ADCs were calculated mathematically, any small experimental error in each phase of the trial could yield the digestibility values beyond the range of 0%–100% [6].

The lipid in CM was digested a bit higher than 100% which was in agreement with the work of Greiling et al. [38]. They determined a CL ADC of 105% for a partially dehulled rapeseed cake in trout but with strpping feces collection methodology. They believed that the interaction among examined diet ingredients led to that result. Digestibilities beyond 0%–100% were also reported by other researchers [31]. The finding for CL ADC in CM was also in line with Hilton and Slinger [39] who measured a value of 93% for that oilseed meal in rainbow trout. The tested IRSBM illustrated apparent CL digestibility of 84% which was less than 96% measured by Sevgili et al. [24]. The variation of lipid digestibility in our work to previous studies could result from either the differences in lipid extraction methodology as mentioned by Bureau et al. [11], or the fish-meal-based reference diet used in that work. According to Bureau et al. [40], the polyunsaturated fatty acids (PUFAs) had a synergistic influence on the digestibility of saturated fatty acids (SFAs). Although they tested beef tallow as a feed ingredient with high levels of SFAs, it is also likely that an interaction among PUFAs in fish meal affected the CL digestibility of SBM. For having a more realistic comparative digestibility assessment among studies, it is recommended to pay attention not only to feces collection methodology but also to the reference diets whether formulated with purified or practical-type feed ingredients.

### 4.3. Digestibility of Organic Matter

OM digestibility is the sum of protein, lipid, and carbohydrate digestibilities. However, OM digestibility has its own relevance with respect to the environmental impact of aquaculture. According to Sugiura and Hardy [5], the higher OM in feces, the superior bacterial fermentation and biological oxygen demand in the sediments of a pond. Therefore, an improved OM digestibility in feed components might create a better rearing environment for the cultured organisms as well as reducing the impact of water bodies receiving the aquaculture effluents. The digestibility of OM content in the tested FeMs in this study ranged from 58% for RegFeM to 88% for the IRFeM. The observed OM ADC for RegFeM was in line with the measured value of 63% for a hydrolyzed feather meal in chinook salmon in sea water [21]. The relative OM ADC of IRFeM to the reference diet was improved with citric acid supplementation which reflected the improved lipid digestibility in that thermochemically hydrolyzed feather meal. The PBM_LA (ash content: 12% DM) had an OM digestibility of 93% which was the best among other tested PBMs. However, the other grade, PBM_50, demonstrated 73% OM ADC. This was comparable to poultry meals (66% and 76%) tested in chinook salmon by Hajen et al. [21]. The minimum observed OM ADC for poultry meal in this study was also higher than the determined 63% for PBM in rainbow trout by other researchers [24]. PPCon showed apparent OM digestibility of 100% which was the highest amongst other tested ingredients. This is in line with high CP and CL digestibilities of this valuable ingredient. The OM in poultry ultra-flash-dried BM was digested by 85% which can be attributed to the CP content in that ingredient. Lipid digestibility for that feed ingredient was determined to be negative. The OM digestibility of BM was higher than the value of 35% which was previously measured in chinook salmon [21]. Technological advances in processing of poultry BM have probably affected protein digestibility and this improved OM ADC in this study rather than previous works. In contrast to the observed high CP and CL digestibilities in the tested CM, that plant ingredient showed only an OM ADC of 65%. It seems likely that the fiber fraction from OM resulted in lower value for OM ADC because fiber is categorized as an indigestible material in almost all fish [6]. Hajen et al. [21] reported OM ADC of 54% for CM in chinook salmon and mentioned high fiber and carbohydrate contents as a reason for the low OM digestibility. In this study, the apparent OM digestibility of 85% for the IRSBM. This finding was relatively higher than values reported previously in salmonids. Glencross et al. [31] observed apparent OM digestibilities of 77% and 73% for rainbow trout and Atlantic salmon, respectively. Likewise, an OM ADC of 73% was determined by Sevgili et al. [24] in rainbow trout. The relatively higher coefficients of apparent OM digestibility for the tested ingredients observed in our study could be caused by the higher content of carbohydrates in the casein-based reference diet used in our work compared to previous studies that used fish meal-based diets as a reference.

### 4.4. Nitrogen and Lipid Retention

Hardy and Barrows [14] mentioned that a practical way to assess the availability of nutrients is to determine the deposition of those nutrients in the whole body of fish over a particular time period as nutrient productive value. Therefore, we calculated the deposition of nitrogen and lipid in fish for tested ingredients under the NPV and LPV, respectively. As all ingredients tested in this study are protein rich, protein content, and protein/energy ratio in the test diets are higher than that of the reference diet (Table 4). Highest nitrogen retention was observed for PBMs followed by PPCon as well as IRSBM, which might reflect a balanced amino acid profile for rainbow trout in these components compared to other ingredients. Lower nitrogen retention was determined for FeMs and BM which was in contrast to their high protein digestibilities. A high positive association between ingredients NPV and their LPV was recognized (*r* = 0.68, *p* = 8.4e^−11^). This correlation supports the hypothesis of protein sparing by lipids [41, 42]. It seems that both ingredients' lipid quantity and quality influenced the protein sparing in the tested ingredients. The concentration of CL in the tested FeMs and BM was almost lower than that in poultry meals. Moreover, the possible rest of poultry skin in FeMs as mentioned by Hertrampf and Piedad-Pascual [43] may have had a negative influence. The saturated and monounsaturated fatty acids could result in lower lipid digestibility and less energy availability in fish [25, 26, 42, 44]. The findings of this study indicated that the refined or low-ash PBM and PPCon as well as IRSBM have a high protein retention in rainbow trout. It can be noted that the inclusion rate in commercial diets should be at the levels that provide the minimum required digestible protein and essential amino acids for the ingredients with poor nitrogen productivity. The needs for digestible energy must then be met by adding high digestible oils. To evaluate the usefulness of feed ingredients for fish in the future studies, the observed findings recommend to measure both the nutrient digestibility and retention of tested feed components.

## 5. Conclusion

The observations with rainbow trout suggest that PBMs, if refined and properly processed, are valuable feed components. Moreover, PPCon is a potential and valuable aquafeed ingredient regarding its high protein and lipid digestibility/retention in rainbow trout. Technological processes can improve protein digestibility of FeMs, converting it from a low-value material, e.g., used as fertilizer, to a high-quality ingredient for aquafeeds. Similarly, the nutritional quality of CM can be improved beyond the current level found in Iran. In summary, various feed resources which are presently not being used for aquafeeds exist in Iran, but appropriate processing is required prior to the large-scale application in feeds for rainbow trout.

## Figures and Tables

**Table 1:** Ingredients of experimental diets (g·kg^−1^ dry matter).

	Run 1		Run 2		Run 3	
Ingredient	Ref^†^	Test diet 1	Ref^†^	Test diet 2	Ref^†^	Test diet 3
Test ingredients 1^‡^	—	298.5	—	—	—	—
Test ingredient 2^⁋^	—	—	—	298.5	—	—
Test ingredients 3^§^	—	—	—	—	—	298.5
Casein	400	280	400	280	400	280
Gelatin	40	28	40	28	40	28
Cellulose^1^	132	92.4	132	92.4	101.2	70.8
Dextrin	90	63	90	63	90	63
Pregelatinized corn starch^2^	107.4	75.2	107.4	75.2	107.4	75.2
Fish oil	150	105	150	105	150	105
Canola oil	49	34.3	49	34.3	49	34.3
L-Arginine^3^	1.9	1.3	1.9	1.3	1.9	1.3
Monocalcium phosphate	15.5	10.9	15.5	10.9	15.5	10.9
Sodium carbonate	—	—	—	—	30.8	21.6
Choline chloride 98%	1	0.7	1	0.7	1	0.7
Vitamin C 35%	0.2	0.14	0.2	0.14	0.2	0.14
Vitamin premix^4^	5	3.5	5	3.5	5	3.5
Mineral premix^4^	3	2.1	3	2.1	3	2.1
TiO_2_	5	5	5	5	5	5
Total	1,000	1,000	1,000	1,000	1,000	1,000

^†^Ref, casein-based semisynthetic laboratory standard diet; ^‡^regular feather meal (RegFeM), GOLDMEHL® feather meal (GoldFeM), Iranian feather meal (IRFeM), poultry by-product meal with minimun 64% crude protein (PBM_64) and canola meal (CM) were tested; ^⁋^its test ingredient was Iranian feather meal but the diet was treated with citric acid (dissolved 0.02 g citric acid, C_6_H_12_O_6_, per 1 g Iranian feather meal and sprayed on that diet or 0.6% diet); and ^§^poultry by-product meal with the maximum 11% ash content as low-ash (PBM_LA), poultry by-product meal with minimum 50% crude protein (PBM_50), poultry protein concentrate (PPCon), poultry ultra-flash dried blood meal (BM), and Iranian soybean meal (IRSBM) were tested. ^1^Provided by Mikro-Technik GmbH & Co. KG, Bürgstadt am Main, Germany; ^2^provided by Kröner-Stärke GmbH, Ibbenbüren, Germany; ^3^provided by Evonik Nutrition and Care GmbH, Hanau-Wolfgang, Germany; and ^4^Vitamin and mineral requirements of fish were met. Provided by Trouw Nutrition Deutschland GmbH, Burgheim, Germany.

**Table 2:** Experimental conditions of the three trials.

	Run 1	Run 2	Run 3
Volume (L)			
Whole system		1,500	
Aquarium		57	
Water inflow rate (L/min)			
Well water inflow into the system		6	
Water flow to each aquarium		3	
Water turnover rate (times/day)			
Whole system		5.8	
Aquaria		75.8	
Water source		Preprocessed well water	
Number of aquaria per diet		4	
Type of aquaria		Rectangular glass, tapered bottom	
Photoperiod (light:dark)		12 : 12 with LED light	
Number of fish per aquarium		15	
Room temperature (°C)		15	
Average water temperature (°C)^†^			
Aquaria	12.8 ± 0.1	12.7 ± 0.1	13.2 ± 0.0
System inflow	13.0 ± 0.4	12.9 ± 0.3	13.0 ± 0.4
System outflow	12.8 ± 0.3	12.8 ± 0.2	13.0 ± 0.3
Dissolved oxygen (mg/L)^†^			
Aquaria	9.9 ± 0.1	9.9 ± 0.2	10.1 ± 0.0
System inflow	10.0 ± 0.4	10.1 ± 0.2	10.1 ± 0.2
System outflow	10.0 ± 0.3	10.1 ± 0.2	10.2 ± 0.2
pH^†^	7.8 ± 0.1	7.9 ± 0.1	8.1 ± 0.1
NH_4_^+^ mg/L^†^	0.20 ± 0.3	0.07 ± 0.0	0.09 ± 0.0
NO_2_^−^ mg/L^†^	0.31 ± 0.3	0.11 ± 0.1	0.23 ± 0.1
NO_3_^−^ mg/L^†^	4.79 ± 1.2	<4.4 ± 1.2	4.5 ± 0.3
Average stocking weight (g)^†^	18.9 ± 1.2	22.7 ± 2.2	12.0 ± 0.9
Initial average total length (cm)^†^	12.3 ± 0.8	14.3 ± 1.1	10.7 ± 0.4
Stocking density (kg/m^3^)	4.7	5.7	3.0
Feeding level (% biomass/aquarium)	1.5	2	2
Acclimation period (days)	5	4	3
Trial period (days)	56	30	56

^†^Reported mean values with standard deviation (±SD).

**Table 3:** Chemical composition analysis in the test ingredients^†^ (g·kg^−1^ dry matter).

Chemical composition	Run 1					Run 2	Run 3				
	FeM			PBM	CM	FeM	PBM		PPCon	BM	IRSBM
	RegFeM	GoldFeM	IRFeM	_64		IRFeM^+^	LA	_50			
Dry matter	960	921	913	938	923	913	927	955	957	928	945
Crude protein	925	947	832	696	379	832	745	544	745	958	491
Crude lipid	69	49	16	118	110	16	120	147	96	19	41
Crude ash	25	19	132	143	74	132	120	334	106	32	92
Organic matter (OM)^⁋^	975	981	868	857	926	868	880	666	894	968	908
Calcium^††^	6	4	n.a.	54	7	n.a.	33	130	2	1	n.a.
Phosphorous^††^	4	2	n.a.	30	13	n.a.	22	69	10	5	n.a.
pH	5.7	6.5	10.1	6.0	6.2	n.a.	n.a.	n.a.	n.a.	n.a.	n.a.
Amino acids^††^											
Alanine	41.0	39.1	39.6	50.0	18.2	39.6	38.0	29.3	36.2	55.3	n.a.
Arginine	61.6	72.8	42.5	54.3	24.5	42.5	54.3	40.2	43.6	50.0	n.a.
Aspartic acid/asparagine	63.0	68.5	57.9	64.1	27.6	57.9	70.7	46.7	52.1	100.0	n.a.
Cysteine (cystin)	50.1	51.1	19.9	6.5	10.3	19.9	26.1	4.3	6.4	12.8	n.a.
Glutamic acid/glutamine	102.1	110.9	94.3	97.8	73.4	94.3	101.1	71.7	98.9	102.1	n.a.
Glycine	67.7	89.1	70.3	97.8	19.8	70.3	63.0	79.3	94.7	37.2	n.a.
Histidine	9.9	7.6	10.3	10.9	11.4	10.3	19.6	10.9	10.6	57.4	n.a.
Isoleucine	41.5	47.8	39.2	28.3	14.7	39.2	38.0	18.5	19.1	43.6	n.a.
Loucine	75.0	82.6	69.3	50.0	27.7	69.3	75.0	33.7	36.2	119.1	n.a.
Lysine	17.0	18.5	11.9	47.8	23.9	11.9	47.8	33.7	37.2	95.7	n.a.
Methionine (methioninsulfon)	5.3	4.3	4.9	14.1	7.9	4.9	12.0	10.9	10.6	11.7	n.a.
Phenylalanine	45.7	48.9	42.2	30.4	15.8	42.2	40.2	18.5	19.1	57.4	n.a.
Proline	77.3	100.0	89.2	60.9	24.7	89.2	64.1	40.2	54.3	41.5	n.a.
Serine	106.3	126.1	61.2	25.0	17.7	61.2	56.5	17.4	27.7	37.2	n.a.
Threonine	44.4	43.5	22.8	25.0	16.8	22.8	35.9	18.5	22.3	46.8	n.a.
Tryptophan	5.2	9.8	6.2	n.a.	5.7	6.2	n.a.	n.a.	n.a.	n.a.	n.a.
Tyrosine	29.1	13.0	19.2	16.3	10.3	19.2	18.5	9.8	5.3	17.0	n.a.
Valine	59.1	39.1	65.7	35.9	18.8	65.7	58.7	21.7	23.4	70.2	n.a.

^†^FeM, feather meal; RegFeM, regular feather meal; GoldFeM, GOLDMEHL® feather meal; IRFeM, Iranian feather meal; PBM_64, poultry by-product meal with minimum 64% crude protein; CM, canola meal; IRFeM^+^, Iranian feather meal diet treated with citric acid (dissolved 0.02 g citric acid, C_6_H_12_O_6_, per 1 g Iranian feather meal and sprayed on IRFeM diet or 0.6% diet); PBM_LA, low-ash (<11%) poultry by-product meal; PBM_50, poultry by-product meal with minimum 50% crude protein; PPCon, poultry protein concentrate or AquaTrac® sol SD; BM, ultra-flash dried poultry blood meal; and IRSBM, Iranian soybean meal. ^⁋^Calculated by subtracting the crude ash content from 1,000. ^††^Adopted from producers' specifications. n.a., not analyzed.

**Table 4:** Chemical composition analysis in experimental diets^†^ (g·kg^−1^ dry matter).

	Run 1						Run 2		Run 3					
	Ref	FeM			PBM	CM	Ref	FeM	Ref	PBM		PPCon	BM	IRSBM
		RegFeM	GoldFeM	IRFeM	_64			IRFeM^+^		LA	_50			
Dry matter	909	910	906	897	915	902	909	896	897	902	888	902	906	873
Gross energy (MJ/kg)	23.4	23.8	23.7	22.9	23.1	22.8	23.4	22.2	23.0	23.4	29.8	23.0	23.4	22.1
Crude protein	434	593	601	553	557	437	434	558	399	502	465	516	568	427
Crude lipid	174	154	144	127	173	175	174	128	183	176	149	169	124	138
Crude fiber	88	68	70	65	69	100	88	64	70	50	50	44	49	71
Crude ash	32	32	30	55	65	44	32	56	52	70	137	67	46	64
Total phosphate	n.a.	n.a.	n.a.	n.a.	n.a.	n.a.	n.a.	n.a.	6.8	10.4	21.8	7.7	6.4	6.7
Calcium	n.a.	n.a.	n.a.	n.a.	n.a.	n.a.	n.a.	n.a.	3.3	11	36.1	3.1	2.7	3.6
Nitrogen-free extract (NFE)^‡^	272	153	155	200	136	244	272	194	296	202	202	204	213	300
TiO_2_	5.4	5.6	5.4	5.4	5.4	5.4	5.4	5.4	5.4	5.3	5.3	5.3	5.3	5.4
Organic matter (OM)^⁋^	968	968	970	945	935	956	968	944	948	930	863	933	954	936
NFE:OM (%)^§^	28	16	16	21	15	26	28	21	31	22	23	22	22	32
pH	4.9	5.0	5.3	7.0	5.3	5.4	4.9	6.5	8.9	7.8	8.1	7.1	7.6	8.0

^†^Ref, casein-based semisynthetic laboratory standard diet; FeM, feather meal; RegFeM, regular feather meal; GoldFeM, GOLDMEHL® feather meal; IRFeM, Iranian feather meal; PBM_64, poultry by-product meal with minimum 64% crude protein; CM, canola meal; IRFeM^+^, Iranian feather meal diet treated with citric acid (dissolved 0.02 g citric acid, C_6_H_12_O_6_, per 1 g Iranian feather meal and sprayed on that diet or 0.6% diet); PBM_LA, low-ash (<11%) poultry by-product meal; PBM_50, poultry by-product meal with minimum 50% crude protein; PPCon, poultry protein concentrate; BM, ultra-flash dried poultry blood meal; and IRSBM, Iranian soybean meal. ^‡^Calculated by subtracting crude protein, crude lipid, crude fiber, and crude ash from 1,000. ^⁋^Calculated by subtracting the crude ash content from 1,000. ^§^Calculated by dividing NFE by OM and multiplying 100. n.a., not analyzed.

**Table 5:** Apparent digestibility coefficients (ADCs) for crude protein (CP), crude lipid (CL), and organic matter (OM) and nutrient productive values in experimental diets and test ingredients in percent (means ± SD).

		Run 1						Run 2		Run 3					
	Experimental feed^†^	Ref	FeM			PBM_64	CM	Ref	FeM	Ref	PBM		PPCon	BM	IRSBM
			RegFeM	GoldFeM	IRFeM				IRFeM^+^		LA	_50			
CP_ADC	Diet	98 ± 0.09^a^	86 ± 1.14^c^	93 ± 0.62^bc^	97 ± 0.10^ab^	95 ± 0.32^abc^	97 ± 0.22^abc^	98 ± 0.17	97 ± 0.07 ^*∗*^	98 ± 0.37^ab^	96 ± 0.16^abc^	89 ± 0.25^c^	99 ± 0.01^b^	93 ± 0.25^ac^	98 ± 0.08^abc^
	Ingredient		73 ± 2.42^a^	88 ± 1.27^ab^	96 ± 0.22^b^	89 ± 0.80^ab^	94 ± 0.81^b^		96 ± 0.15		93 ± 0.38^abc^	73 ± 0.68^b^	100 ± 0.06^a^	87 ± 0.51^bc^	97 ± 0.24^ac^

NPV^‡^	Diet	46 ± 6.97^b^	29 ± 8.92^ab^	33 ± 3.72^ab^	23 ± 6.50^a^	46 ± 7.69^b^	40 ± 11.53^ab^	45 ± 7.74	25 ± 1.62 ^*∗*^	39 ± 1.75^ab^	40 ± 0.61^a^	35 ± 1.52^ab^	38 ± 2.93^ab^	31 ± 1.53^b^	38 ± 1.95^ab^
	Ingredient		11 ± 18.76^ab^	18 ± 7.75^ab^	−6 ± 14.48^a^	46 ± 19.00^b^	24 ± 42.44^ab^		0.34 ± 3.64		40 ± 1.31^a^	27 ± 4.11^bc^	37 ± 6.60^ab^	23 ± 3.06^c^	37 ± 5.71^ab^

CL_ADC	Diet	94 ± 0.67^abc^	83 ± 1.18^c^	88 ± 0.64^ac^	90 ± 0.14^abc^	94 ± 0.73^ab^	95 ± 0.35^b^	89 ± 0.98	87 ± 0.54	90 ± 1.24^ab^	92 ± 0.77^a^	89 ± 1.22^ab^	91 ± 0.50^ab^	85 ± 1.43^b^	89 ± 1.73^ab^
	Ingredient		24 ± 8.08^ab^	39 ± 5.97^ab^	6 ± 3.65^a^	98 ± 3.23^b^	101 ± 1.67^b^		55 ± 13.97		99 ± 3.51^a^	87 ± 4.78^ab^	95 ± 2.71^ab^	−39 ± 33.82^b^	84 ± 19.73^ab^

LPV^‡^	Diet	81 ± 10.1^a^	55 ± 14.3^a^	66 ± 6.8^a^	60 ± 8.0^a^	76 ± 13.7^a^	67 ± 14.5^a^	50 ± 7.6	22 ± 8.5	74 ± 1.4^a^	76 ± 1.0^a^	68 ± 3.2^a^	74 ± 7.0^a^	67 ± 8.1^a^	70 ± 4.3^a^
	Ingredient		−94 ± 98.6^ab^	−54 ± 63.9^ab^	−459 ± 208.0^a^	58 ± 60.9^b^	17 ± 68.1^b^		−669 ± 220.3		85 ± 4.8^a^	50 ± 12.5^a^	72 ± 38.3^a^	−94 ± 190.0^a^	22 ± 49.3^a^

OM_ADC	Diet	80 ± 0.58^ab^	73 ± 2.06^a^	79 ± 0.88^ab^	82 ± 0.19^b^	80 ± 0.45^ab^	75 ± 0.13^ac^	76 ± 1.26	81 ± 0.46 ^*∗*^	79 ± 1.5^ad^	83 ± 0.30^c^	78 ± 0.36^d^	85 ± 0.21^b^	81 ± 0.54^e^	81 ± 0.40^ae^
	Ingredient		58 ± 6.81^bd^	77 ± 2.91^a^	88 ± 0.71^c^	81 ± 1.64^ac^	65 ± 0.46^d^		92 ± 1.67		93 ± 1.04^c^	73 ± 1.60^d^	100 ± 0.72^b^	85 ± 1.76^e^	85 ± 1.38^ae^

^†^Ref, casein-based semisynthetic laboratory standard diet; FeM, feather meal; RegFeM, regular feather meal; GoldFeM, GOLDMEHL® feather meal; IRFeM, Iranian feather meal; PBM_64, poultry by-product meal with minimum 64% crude protein; CM, canola meal; IRFeM ^+^, Iranian feather meal diet treated with citric acid (dissolved 0.02 g citric acid, C_6_H_12_O_6_, per 1 g Iranian feather meal and sprayed on IR diet or 0.6% diet); PBM_LA, low-ash (<11%) poultry by-product meal; PBM_50, poultry by-product meal with minimum 50% crude protein; PPCon, poultry protein concentrate or AquaTrac® sol SD; BM, ultra-flash dried poultry blood meal; IRSBM, Iranian soybean meal. ^‡^Nitrogen productive value (NPV); lipid productive value (LPV). The means within one line in each run (1&3) not sharing a superscript letter (a, b, c, d, e, ab, ac, ae, bc, bd, abc) are significantly different (*p* < 0.05). Values with asterisks are considerably different (*p* < 0.05) in run 2 only. Values for the ingredient IRFeM^+^ were not compared to other values.

## Data Availability

All the data in the manuscript are available upon request.
